# Rumenotomy

**Published:** 1882-04

**Authors:** J. Lindsay


### II.—RUMENOTOMY.

REPORTED BY J. LINDSAY, D.V.S.

On July 18th I was first called to attend a cow attacked with the following symptoms:

This unfortunate animal appeared to be suffering from impactione of th rumen.

Physical examination gave the following result: When the flank was pressed upon, over the region of the rumen, there was no perceptible indentation after the pressure was removed. Similar symptoms are met with in connection with a paralysis of the rumen, but in such cases a depression remains after removal of the pressure. From this I diagnosticated positively an over-distention of the rumen, and was led to believe the difficulty might be relieved by a brisk cathartic, and therefore ordered the following:

℞

Magnesia Sulphatis, et.

Sodii Chloridium, āā, ℥ viii.

Pulsis Zingeberis, ʒ ii.

Nuces Vomicæ, ʒ iii.

Aquæ, q. s. a. d., ℈ viij.

Mic. sig. at one drench.

This was to be followed by enemas of strong soap-suds, three times a day, and the animal to be allowed plenty of water to drink.

On the following day I found the cathartic had not acted, but the cow appeared brighter, and I concluded to wait another day before resorting to any operation. Ordered the enemas to be kept up, and as the animal was quite thirsty, took advantage of this fact to heip the cathartic by a greater bulk of fluid, thinking that, at the end of twenty-four hours more, the cathartic would act.

On the 20th the cathartic had not acted; the animal was dull, had lost her appetite, was no longer thirsty, had a staggering gait and was partially paralyzed in the posterior extremities.

Upon examination I decided that there was positive pittng of the rumen, and from this symptom felt fully convinced that I must operate immediately or the life of the animal would be lost.

Informing the owner of this fact and gaining his consent, I had the animal placed in a stanchion, with her right side against the wall, and strapped the posterior limbs firmly together.

I opened the abdominal cavity by an incision midway between the ilium and the last rib. The incision was commenced 7.5 centimetres external to the transverse process of the fourth lumbar vertebra, and extended outward and downward for 18 centimetres, cutting through the integument fascia and external and internal oblique muscles, the transversalis muscle and fascia, thus reaching the peritoneal coat, which was carefully cut through. In this way the centre of the rumen was exposed to view, and bulged out through the external wound. The rumen was carefully opened, and the edges carefully sewed to those of the external incision, to prevent any of the contents of the rumen falling into the peritoneal cavity.

This much having been successfully accomplished, I proceeded to remove the contents, which were very solid, and had to be broken down by the hand before any portion could be removed. I removed in all about two bushels of this impacted matter and carefully cleansed the parts with carbolized warm water (1 to 40).

The lips of the wound in the rumen were brought together in a way to secure perfect coaptation of the anatomical structures, especially the peritoneal coat, and temporarily held in this position by interrupted, carbolized cat-gut sutures.

The external incision was next brought together, and held in position by the same kind of sutures. The wound was then perfectly covered in by a large pitch plaster, thus excluding as much air as possible, and preventing the access of flies. Administered:

℞

Spiritus Etheris Nitrosi, ℥ vi.

Aquæ, q. s. ad., O i.

Mic. sig. pr. r. n.

I also had her covered with a fly blanket, and administered as a tonic:

℞

Aquæ Ammonia, et.
Tincture Zingeberis, et.
Tincture Gentian, āā, ℥ vi.

Mic. sig. ℥ iij. t. i. d. in a pint of gruel.

21st. The animal appeared somewhat brighter, had eaten some hay and drank a bucket of water. Diarrhœa set in, and the fæcal discharges were very offensive. Cut off water supply and gave flour gruel, medicated with

℞

Tincture Opii, et.

Creta Preperata, āā, ℥ ii.

Sodæ Hyposulphatis, ʒ iv.

Mic. sig. in gruel.

22d. Animal much improved; diarrhœa checked; fæces nearly normal; appetite good; had laid down during the night. Stopped gruel and gave two quarts of steamed oats, with a little hay. Tonic still continued.

23d. Patient discharged, with orders to add small quantity of green food with the regular diet.

August 2d. Was again called in, and examined the wound, which was swelling very badly. The reason of this was that the plaster had slipped down, leaving a small hole at the top of the wound, into which flies had found their way, and it was alive with maggots. After removing the whole of the plaster, I found the stitches had all been torn out and the wound discharging very fœtid pus.

The wound in the rumen, however, had fully healed, and digestion was being performed perfectly. I mixed ℥ ii ot Calvert's carbolic acid No. 2 in one pint of water, and had the wound thoroughly syringed with the same every three hours. No attempt was made to bring this granulating wound together with sutures, but it was left to close by granulations from the bottom. Continued the use of the carbolic acid solution and kept it covered with a piece of muslin, saturated with the same solution, several layers of cloth, saturated with the carbolic acid solution, which was glued to the superior ridge of the back with pitch; in this way it was kept constantly covered. Over this dressing an abdominal bandage was applied and the cow covered with a fly blanket. In this way she was kept very nearly under a perfect antiseptic influence.

Sept. 5. The animal was perfectly cured.

I consider the results of this case as a fair test of the carbolic acid as an antiseptic dressing in veterinary surgery, and one which can be resorted to in an emergency.

This wound certainly had fallen into as bad a shape as we could expect to meet with, for it was filled with maggots and in a state of rapid decomposition.

The way in which the wound got into this very bad condition was by the neglect of the owner, who turned her out to pasture and failed to closely watch the progress of the wound. Several weeks after the wound had perfectly healed and there was no perceptible cicatrix visible.

HUNTINGTON, L.I., 1882.

[This case is certainly one of considerable interest, forcibly showing the value of antiseptic treatment of wounds in veterinary surgery when practically applied. The results were certainly all that any surgeon could desire. The way, also, in which the dressings were held in position is novel, and one that will probably prove of advantage and worthy the attention of veterinary surgeons.

The use of pitch, however, as a surgical dressing for animals is not new, but the manner in which it was applied in this case, I think, is entirely so. Antiseptic dressings have wrought wonderful changes in human surgery, and have been too long and too often neglected in comparative science. Here, however, we have a clear demonstration of its power, and a warning to all not to neglect its constant use in the future. If, then, the older methods of treatment allow the wounds of the unfortunate animal to become filled with maggots and decomposing matter, and modern treatment will effectually prevent such horrible conditions, it certainly is high time that the newer and more advanced ideas took the place of ancient customs. What condition of a wound could be more appalling to any sensitive or kind-hearted nature than that of one alive with maggots and one giving forth the most offensive odors from decomposition of the tissues. This case ought to be a warning to every veterinary surgeon, and always keep him on his guard against such conditions.—Ed.]

